# Water-Mediated Excited State Proton Transfer of Pyranine–Acetate in Aqueous Solution: Vibrational Fingerprints from Ab Initio Molecular Dynamics

**DOI:** 10.1021/acs.jpca.1c00692

**Published:** 2021-04-26

**Authors:** Maria Gabriella Chiariello, Umberto Raucci, Greta Donati, Nadia Rega

**Affiliations:** † Dipartimento di Scienze Chimiche, Università di Napoli Federico II, Complesso Universitario di M.S. Angelo, via Cintia, I-80126 Napoli, Italy; ‡ Centro Interdipartimentale di Ricerca sui Biomateriali (CRIB) Piazzale Tecchio, Largo Barsanti e Matteucci, I-80125 Napoli, Italy

## Abstract

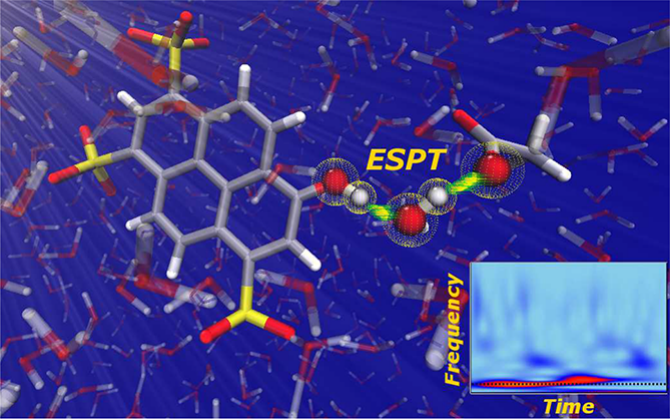

In this work, we simulate the excited state proton transfer (ESPT) reaction involving the pyranine photoacid and an acetate molecule as proton acceptor, connected by a bridge water molecule. We employ ab initio molecular dynamics combined with an hybrid quantum/molecular mechanics (QM/MM) framework. Furthermore, a time-resolved vibrational analysis based on the wavelet-transform allows one to identify two low frequency vibrational modes that are fingerprints of the ESPT event: a ring wagging and ring breathing. Their composition suggests their key role in optimizing the structure of the proton donor–acceptor couple and promoting the ESPT event. We find that the choice of the QM/MM partition dramatically affects the photoinduced reactivity of the system. The QM subspace was gradually extended including the water molecules directly interacting with the pyranine–water–acetate system. Indeed, the ESPT reaction takes place when the hydrogen bond network around the reactive system is taken into account at full QM level.

## 1 Introduction

In excited state proton transfer (ESPT) reactions, a compound reacts to the absorption of radiation by releasing protons.^1,2^ The newly formed deprotonated species usually exhibit different spectroscopic properties in terms of absorption and fluorescence spectra and vibrational signatures.^3,4^ For this reason, these compounds represent a promising class of light-sensitive molecules for applications in the field of biological imaging, as optoelectronic devices, and as fluorescent probes in complex environments.^5−7^ The so-called photoacid molecules belong to a large family of organic compounds known to give the excited state proton transfer reaction.^8−11^ Generally, in the ground electronic state (S_0_), the proton transfer reaction is thermodynamically and kinetically unfavorable or extremely slow, and the photoacid remains in its protonated form. The absorption of UV–vis radiation leads the molecule to an electronic excited state, inducing a deep rearrangement in its electronic structure.^12−14^ The electronic excitation gives rise to a new reactivity, dramatically different from the ground state behavior, which allows the release of the proton to a nearby solvent molecule or to a base if present in solution.^15−19^

**Figure 1:**
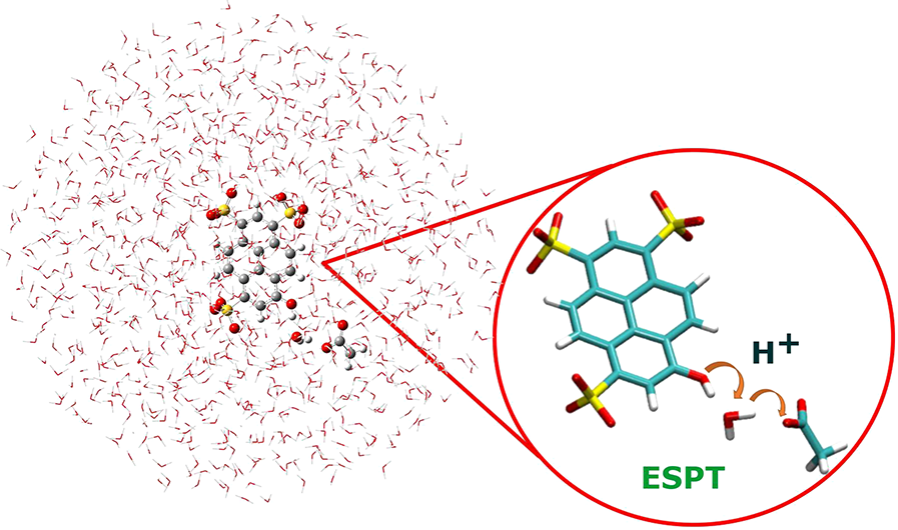
*Pyranine–water–acetate* in aqueous solution: the pyranine–water–acetate system is treated at the QM level (the B3LYP/6-31g(d,p) level of theory is used for both the ground and excited state simulations), while the remaining explicit solvent molecules are modeled by molecular mechanics, according to the TIP3P model.

The 8-hydroxypyrene-1,3,6-trisulfonate (HPTS) or pyranine (Figure 1) is one of the most popular photoacids, which is used as paradigm case to study the elementary steps of the ESPT process.^12,20−23^ It has been classified as a weak photoacid, because of the slow ESPT kinetics compared to other photoacid molecules.^24^ Indeed, while the strongest photoacid recognized so far transfers a proton to a nearby solvent molecule on the subpicosecond time scale (about 100 fs),^25,26^ the shorter kinetic time constants for the ESPT of pyranine in pure water solution have been reported to be about 3 and 90 ps.^8,27,28^ Nevertheless, when a base like acetate is present in solution the reaction becomes faster (kinetic time constants of 300 fs, 1 ps, and 6 ps ).^29,30^ Depending on the acetate concentration, the ESPT proceeds with a direct proton transfer from pyranine to acetate or through the bridge of one or more water molecules linking the acid–base couple.

Femtosecond stimulated Raman spectroscopy (FSRS) experiments revealed a complex vibrational activity underling the nuclear relaxation of the HPTS chromophore in aqueous solution.^31−34^ Specifically, the electronic excitation activates some low frequency (<1000 cm^–1^) vibrational modes having a lifetime on the picoseconds time scale. These vibrational modes characterize the photoexcited HPTS also when the acetate participates to the EPST reaction. Indeed, the presence of the base does not lead to any shift in the electronic excitation (404 nm),^29^ and the nature of the frontier orbitals is unaltered (see Figure S1).

According to the experimental evidence, the ultrafast component of the ESPT kinetic (300 fs) is assigned to the direct ESPT from HPTS to the acetate.^30,35^ In these conditions, with a base in close proximity of the pyranine acid group, the ESPT may proceed barrierless if the acid–base complex is structurally optimized and well oriented already in the ground state. The ultrafast rate suggests that the directly hydrogen-bonded complex is not responsible of the vibrational activity detected on longer time scale.^30^ The water-mediated ESPT mechanism involving the HPTS–acetate couple is an interesting scenario. The ESPT rate is correlated to the size of the solvent-separated HPTS–acetate complex and to the configuration of the water molecules between the acid–base pair.^9^ Despite the fact that the presence of acetate in solution makes the ESPT reaction faster, the HPTS molecule has to rearrange its nuclear structure to optimize the interaction with the proton acceptor (water molecule(s)) through the activation of the characteristic low frequency vibrational modes. This step is preparatory for the subsequent ESPT reaction.^3,4,36,37^ The theoretical modeling of this process would provide physical–chemical insights into the photoinduced water-mediated acid–base reactions.^38−42^

In this work, we explored the ESPT between pyranine and acetate in water using quantum/molecular mechanics (QM/MM) excited state ab initio molecular dynamics. The aim of the work is 2-fold. We first focused on finding the optimal conditions to simulate the proton shuttle from the HPTS to acetate through the water molecule. In particular, our simulations show that the correct solvation at full QM level of the reaction main actors is mandatory to stabilize the excess proton, present as hydronium, between HPTS–acetate pair. Then, the vibrational dynamics leading to the ESPT event has been dissected. The difficulty in reproducing the characteristic vibrational modes underling the excited state dynamics relies on the fact that the conventional static approach for solving the vibrational problem requires the localization of a minimum energy structure on the potential energy surface.^43,44^ Therefore, it is prohibitive for systems in a complex environment. A promising approach to address the issue of capturing the time evolution of the vibrational modes in the nonequilibrium regime has been recently proposed.^45^ Here, the normal modes are evaluated along the excited state trajectory by an instantaneous analytical Hessian evaluation.

On the other hand, we developed a novel vibrational analysis, that combines the extraction of normal modes from ab initio molecular dynamics trajectories^46,47^ with the wavelet transform,^48^ allowing a direct comparison with the experimental signals. The vibrational analysis is performed *a posteriori*; therefore, no additional Hessian evaluation is required. The method has been also recently extended to the study of the far-from equilibrium phenomena,^49−52^ like the vibrational relaxation using as a pivotal application the HPTS chromophore in water solution.^53^ We successfully reproduced the main vibrational modes of the HPTS chromophore, appearing in the first picosecond after the electronic excitation. The same procedure is here applied to the more complex case of HPTS undergoing the proton dissociation. The composition of the two main low frequency vibrational modes, ring wagging (108 cm^–1^) and ring breathing (190 cm^–1^), suggests their key role in optimizing the structural arrangement between the proton donor–acceptor pair. In particular, the breathing mode is responsible for the HPTS skeleton stretching; moreover, the intermolecular stretching of the heavy atoms involved in the proton transfer reaction, makes the proton donor–acceptor couples tighter in the excited state. The ring wagging modulates the orientation of the proton transfer couples due to an out-of-plane component localized on the HPTS phenolic moiety. This induces in the excited state the oscillation around planarity of the intermolecular and intramolecular phenolic dihedral angles.

These results, obtained for an excited state proton dissociation, are a further demonstration that our protocol allows to accurately rationalize the photoinduced nuclear dynamics in terms of vibrational modes. This allows a direct comparison with the experiments and a clear link between the proton transfer dynamics and the underlying vibrational activity. The discussion of the results is organized as follows: In Section 3.1, the ground state equilibrium solvation around the active site is presented. Section 3.2 is dedicated to the simulation of the excited state proton transfer reaction with different QM/MM partitions, whereas the vibrational analysis is finally discussed in Section 3.3. Computational details of the simulations are the subject of the next section.

## 2 Methods

### 2.1 Simulation Details

A cluster was built up including hydrogen-bonded pyranine, water and acetate. The molecular system was placed at the center of a sphere of a radius 19 Å including 1027 water molecules (Figure 1). The implicit and structureless solvent surrounding the explicit sphere was accounted for in the energy potential and completed the hybrid explicit/implicit solvation model. More specifically, nonperiodic boundary conditions accounted for the interactions of both electrostatic and dispersion–repulsion nature between the explicit molecular system and the implicit bulk solvent.^54−56^ The solvent molecules were explicitly represented by the TIP3P water model, while the implicit bulk solvent was represented by the polarizable continuum model in its conductor-like version.^57,58^ The explicit system itself is treated at different levels of theory according to the hybrid QM/MM ONIOM extrapolative method employing an electronic embedding scheme.^59−65^ In particular, the *pyranine–water–acetate* system is described by DFT and TD-DFT in the ground and excited electronic state, respectively, by adopting the global hybrid B3LYP functional and the 6-31g(d,p) basis set. The obtained energy potential ruled the AIMD simulations in both the ground and the first singlet excited state. The ground state sampling was performed by means of the atom-centered density matrix propagation (ADMP) method.^42,66−68^ After 5 ps of equilibration, the trajectory was collected for 10 ps with a time step of 0.2 fs, keeping a constant temperature of 298 K. Excited state trajectories were collected through Born–Oppenheimer ab initio dynamics, with excited state energies and gradients computed on-the-fly by TD-DFT in its linear response formalism.^69−71^ A total of five excited state trajectories were collected, sharing the same starting structure, but employing different QM/MM partitions. The first excited trajectory (*TRAJI*) with the same partition adopted in the ground state sampling, with the *pyranine–water–acetate* system treated at QM level, was considered as reference. Then, the QM region was extended including one, two, three, and five water molecules surrounding the reactive cluster. The starting structure has been chosen in order to be representative of the ground state equilibrium solvation. All the calculations were carried out with Gaussian16 suite program.^72^ All the molecular dynamics simulations were performed on a single node (16 cores), requiring a total of ∼70 000 core-h.

### 2.2 Time-Resolved Frequency Multiresolution Analysis

We adopted a protocol recently introduced by us,^53^ which combines the extraction of generalized vibrational modes defined from ab initio molecular dynamics with a time-resolved analysis based on the wavelet transform.^73,74^ Our approach works as follows. The assumption is that, at any temperature, 3*N* generalized molecular modes **Q** can be defined as vibrational modes whose velocities are uncorelated to each other.^73^ They can be obtained by diagonalizing the **K** matrix of the mass weighted atomic velocities **q̇** with elements
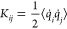
1where *i* and *j* run over the 3N atomic coordinates and ⟨...⟩ indicates the average over the time.^46,47,75,76^

The columns of the transformation matrix **L** are composed of the eigenvectors of **K**. The modes velocities vectors **Q̇**(*t*) are derived by the projection of the mass weighted atomic velocities along the modes and vibrational frequency values can be obtained by Fourier transforming the corresponding autocorrelation functions.

The definition of generalized modes **Q**, unlike that of normal and quasi-normal ones,^77,78^ does not require a quadratic form of the potential, hence these collective coordinates correspond to molecular motions intrinsically anharmonic, showing anharmonic frequencies and coupling to other vibrations.^79^ This methodology has been successfully adopted for the vibrational analysis of molecular systems at the equilibrium, which can be applied to steady-state vibrational spectra.^46−48^ The procedure has been then extended to the analysis of far from equilibrium processes, specifically the transient vibrational signals activated in relaxation processes at the electronic excited state (ES).^53^ During the relaxation, the time evolution of generalized modes **Q**_*ES*_ in the excited state can be obtained from mass weighted atomic velocities **q̇**_*ES*_ extracted and averaged from ES trajectories, according to the projection
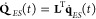
2where **L**^T^ is the transpose of **L**. Here we assume that the modes composition obtained in the ground state (given by **L**^T^) still hold in the excited state, as long as the relaxation has not led to a new arrangement of forces among nuclei and, as a consequence, to a new normal modes composition. This approximation is reasonably true in the ultrafast part of the relaxation and in proximity of the Franck–Condon region. The knowledge of relaxation times from experimental time-resolved spectra can also assist and validate the choice of this approach.

To obtain the vibrational frequency values along the time, we adopted a multiresolution vibrational analysis based on the wavelet transform (WT).^80−85^ We use WT to obtain transient vibrational signals corresponding to the **Q̇***_ES_*(*t*) modes extracted from AIMD. We adopt the continuous WT expression
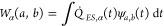
3where α runs over the 3*N* generalized modes. In a manner similar to the quantum mechanical Hessian-based solution of the vibrational problem, 6 (or 5 in linear molecules) of the 3*NQ*_α_ generalized coordinates correspond to translational and rotational modes. Translations and rotations are projected out of the coordinates and momenta during the molecular dynamics trajectories. In this way, time dependent signals **Q̇**_*ES*_(*t*) are analyzed and decomposed in terms of wavelet basis ψ_*a*,*b*_. These are obtained from a so-called mother wavelet by dilatation and translation.
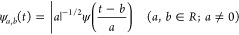
4

We chose the Morlet function as the mother wavelet. The scale parameter *a*, proportional to the inverse of frequency, regulates the dilatation and contraction of the mother wavelet and extracts the different frequencies hidden in the time-dependent signal. On the other hand, the translation of the wavelet basis, ruled by the *b* parameter, ensures the localization of the frequencies in time domain. We plot the magnitude square of the transform |*W*_α_(*v*,*t*)|^2^ as the intensity of the instantaneous frequency contribution to the signal.^49,50^ As final result, we obtain the power spectra of the generalized modes velocity **Q̇**_*ES*_, by retaining localization of each signal in both time and frequency domain. This approach allows one to monitor characteristic photoinduced vibrational dynamics in excited molecules.

## 3 Results and Discussion

### 3.1 Equilibrium Microsolvation from Ground State Sampling

**Figure 2:**
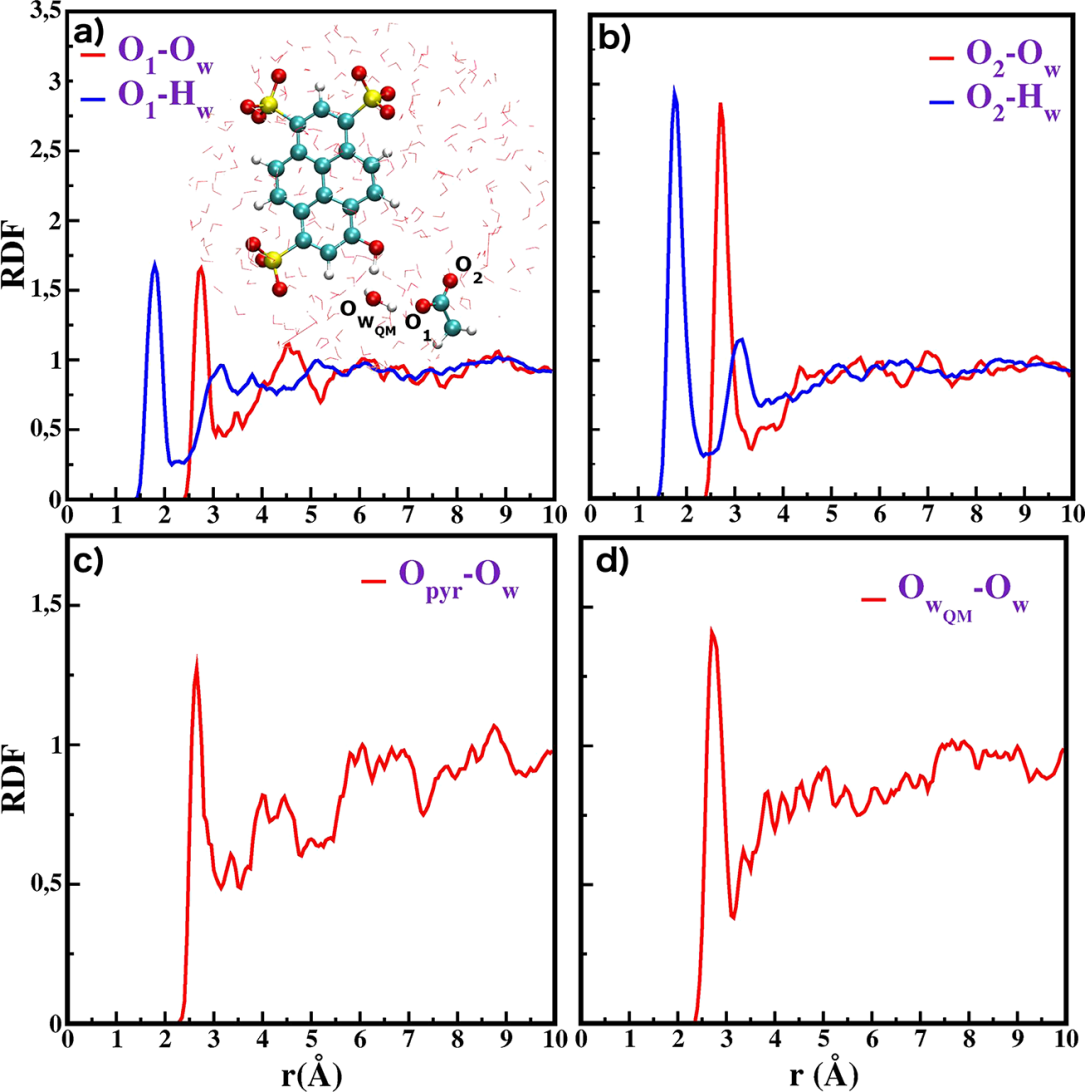
(a) RDFs between O_1_ of acetate and the TIP3P water molecules, (b) RDFs between O_2_ of acetate and the TIP3P water molecules, (c) RDF between the phenolic oxygen of pyranine O_*pyr*_ and the TIP3P water molecules, and (d) RDF between the oxygen of the water belonging to the QM cluster W_*QM*_ and the TIP3P water molecules.

Our analysis begins characterizing the microsolvation around the *pyranine–water–acetate* system (the QM reactive site) at the equilibrium in the ground electronic state. The *pyranine–water–acetate* system lies at the center of a sphere of water molecules explicitly treated, as depicted in Figure 1. The explicit solvent molecules are free to establish hydrogen bond interactions with several solvation sites of the *pyranine–water–acetate* core. The oxygen atoms of acetate are labeled O_1_ and O_2_, indicating the oxygen bound directly to the QM and surrounding waters, respectively (see inset of Figure 2a). The peaks of the acetate oxygen–water oxygen (O_1_–O_*w*_ and O_2_–O_*w*_) radial distribution functions (RDF) are reported in Figure 2a and b. They correspond to the first solvation shells of the acetate molecule and are centered at 2.76 and 2.75 Å for O_1_–O_*w*_ and O_2_–O_*w*_, respectively. The peaks of the O_2_–O_*w*_ are clearly higher, indicating that a greater number of water molecules is included in the first solvation shell. Indeed, integration of the first peak suggests that O_1_ and O_2_ are solvated on average by 1.5 and 2.5 water molecules, respectively. The structuration of the solvent around the phenolic oxygen of pyranine (O_*pyr*_) and the water (O_*WQM*_) belonging to the QM region, is instead described by RDFs shown in Figure 2, parts c and d, respectively.

On average, one water molecule is hydrogen bonded to O_*pyr*_ and . In particular, the peak centered at 2.65 Å for the O_*pyr*_–O_*w*_ distance suggests that a strong hydrogen bond is established between the solvent molecule and the phenolic oxygen of pyranine. As we will discuss later, the solvating water molecule is fundamental in assisting the proton transfer, due to its ability to stabilize the negative charge of the phenolic oxygen during and after the dissociation of the proton. In the same way, the bridge QM water (W_*QM*_) participates in the surrounding hydrogen bonds network playing the role of both acceptor and donor.

The proton transfer event is not observed during the ground state sampling, and the solvent structuration here described is characteristic of the ground state equilibrium. Upon excitation, the dissociation of the proton leads to a redistribution of the electronic density and the water molecules around the *pyranine–water–acetate* system play the key role of stabilizing reactants, transition state, and products. ESPT simulations adopting different sizes of the QM region have been performed and analyzed to disentangle all the effects in play (polarization, electrostatic interactions).

### 3.2 The ESPT Reaction: Effect of the QM/MM Partition

In this section, we discuss the simulation of the ESPT reaction in the *pyranine–water–acetate* system. A proton shuttle occurs from pyranine to acetate via a hydrogen-bonded water bridge. When acetate is present in water solution, the ESPT reaction is accelerated, especially if the base is in close proximity to the phenolic acid group of pyranine. In particular, the fastest detected time constants of 350 and 1000 fs were associated with the direct or mediated by a bridge of few water molecules proton transfer, respectively.^30,35^ The ESPT kinetics is however strongly dependent on the nuclear structure of the starting configuration, i.e., the intermolecular distances of the proton donor–acceptor pair and their relative orientation, in addition to the solvation around the reactive core.^3,4,86^ If the starting nuclear configuration is structurally prepared for the ESPT reaction, it reasonable to expect an ESPT in the subpicosecond time scale.

**Figure 3:**
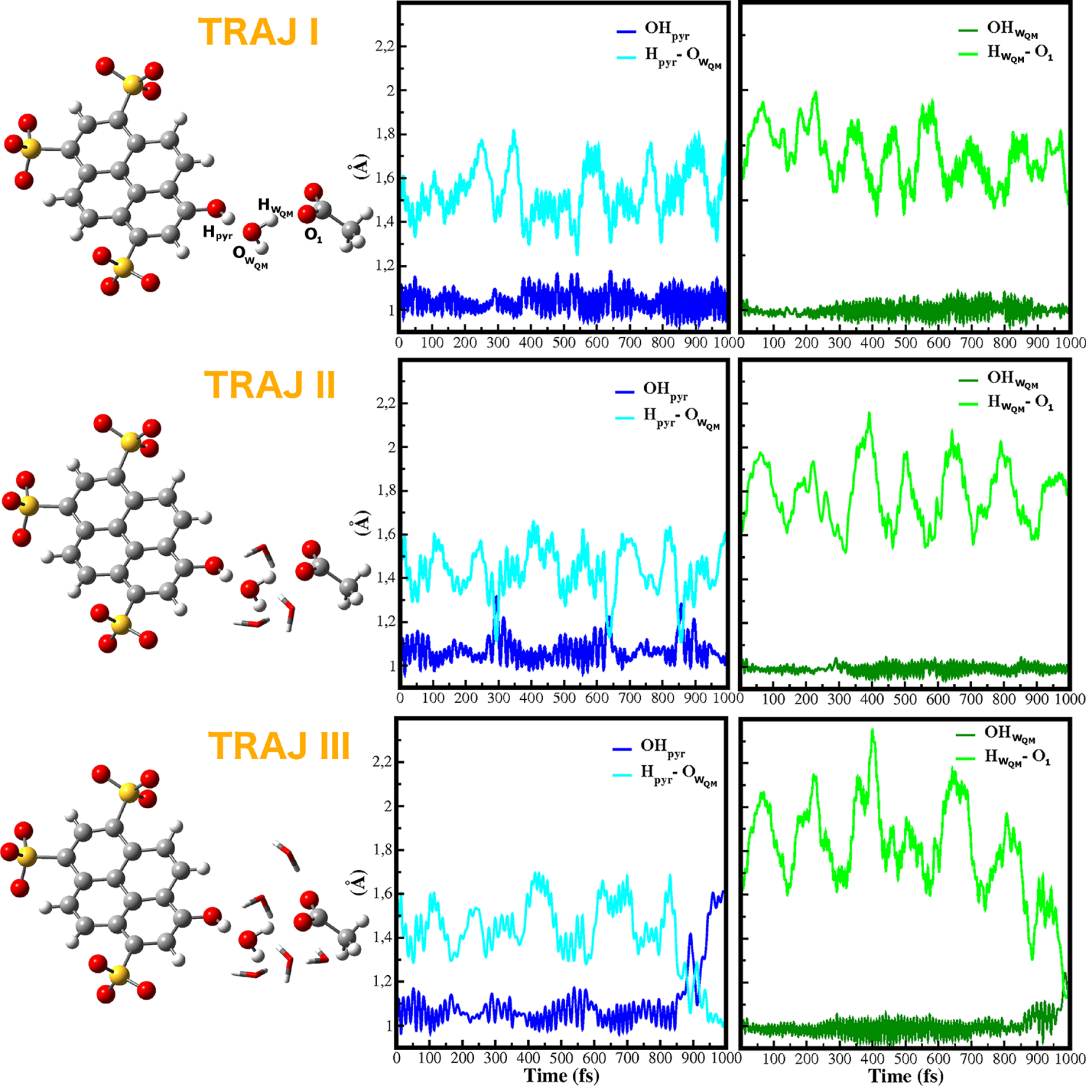
Excited state trajectories for three QM/MM partitions. Left: Three (*TRAJII*) and five (*TRAJIII*) water molecules depicted in licorice representation are included in the QM region, in addition to the *pyranine–water–acetate* system (*TRAJI*). Right: Time evolution of the OH_*pyr*_, , and H_*pyr*_–, –O_1_ parameters on the excited state.

Moreover, the solute–solvent interactions have to be accurately taken into account to provide a reliable description of the ESPT reaction. Understanding how accurate is the treatment of the QM/MM boundary is not a simple task. In a recent work, we explored the ESPT reaction of a superphotoacid to the solvent by means of the same QM/MM hybrid scheme.^86^ Despite the ultrafast reactivity (100 fs), we found that the ESPT is assisted by the solvation dynamics of the water molecules belonging to the first and second solvation shell of the first accepting water molecule. Additionally, we found that this event can be reproduced only when solvation shells around the proton are taken into account at the full QM level. Here, the proton is not free to diffuse through the solution, being the acetate the final destination. Nevertheless, the simulation of the proton motion through the *pyranine–water–acetate* triad may also require a proper extension of the QM space. On this ground, we run five excited state trajectories (see Figure 3, Figures S2 and S3) featuring different QM/MM partitions. The first one (*TRAJI*) has the same ground state layout, namely that *pyranine–water–acetate* are treated at the QM level. In Figure S2, we compare significant structural parameters in the ground and excited state. The comparison shows that the hydrogen bond between pyranine and water oscillates around lower values on the excited state (from 1.6 Å in S_0_ to 1.4 Å in S_1_). On the other hand, the proton donor–acceptor pair involved in the subsequent proton transfer (water–acetate pair) undergoes slight changes on average, with values of the –O_1_ bond of about 1.78 and 1.70 Å in the ground and excited states, respectively. The electronic excitation makes the first proton donor–acceptor pair immediately stronger and tighter. Nevertheless, with a QM/MM boundary restricted to the pyranine–water–acetate system, no proton transfer event is observed. Furthermore, no remarkable differences are detected increasing the size of the QM region by including the water molecule hydrogen bonded to the phenolic oxygen of pyranine (Figure S3). The pyranine remains protonated during the simulation time of one ps.

In *TRAJII* (Figure 3 middle panel), the QM region includes, in addition to the water solvating the pyranine, also a couple of solvent molecules around the central water. We observe short proton jumps from pyranine to water along the trajectory. In particular, at times of 300, 620, and 850 fs the proton moves briefly to the water, but a permanent ESPT leading to a stable anionic pyranine does not occur. Moreover, the water–acetate couple does not seem to be affected by the proton hops between pyranine and water. The central water acts both as proton acceptor and donor toward pyranine and acetate respectively, and its solvation at a QM level seems to promote the first proton transfer event but not the final proton transfer toward acetate. The final trajectory (*TRAJIII*) features the largest QM partition, which includes two more water molecules around the oxygen of acetate (Figure 3). The excited state dynamics shows that, at 800 fs, the proton moves from pyranine to water, with the subsequent formation of an hydronium ion. This entity has a lifetime of about 100 fs after that the proton jumps to the acetate at the time of 1 ps. It turns out that the ESPT proceeds with a nonconcerted mechanism through the formation of the hydronium. An analysis of Figure 3 shows that until 800 fs the intermolecular –O_1_ distance in the excited state does not oscillate around values lower than the ground state. The water–acetate pair starts to approach as soon as the proton moves from pyranine to water and the hydronium is formed. The water molecules promoted to the QM region remain hydrogen bonded to the *pyranine–water–acetate* system (see Figure S4). The analysis of these trajectories shows a clear trend toward promoting the ESPT reaction with increasing the number of solvent molecules included in the QM/MM partition. The first important effect is achieved with the inclusion of the solvation shell around the central water (*TRAJII*) when short proton hops are observed within the pyranine–water couple. The difference is that the oxygen atoms of the solvent, now included in the QM region, are described accurately by means of the two electron pairs and no longer with the MM charges. That allows for having optimal H-bond orientation and interaction with the first proton-acceptor atom, i.e., the oxygen of the central water. On this way, the inclusion of the solvent water around the second proton-acceptor atom (oxygen of acetate) makes possible the simulation of the ESPT. We performed also an excited state simulation (Figure S5) which starts from a different initial configuration but shares the same QM/MM partition, i.e., five water molecules hydrogen bonded to the *pyranine–water–acetate* system are included in the QM region. Here, the ESPT is also detected within 1 ps. Despite the computational demand of running excited state simulations does not allow to collect a satisfactory statistics, our results should support the general reliability of the adopted QM/MM layout.

### 3.3 Vibrational Fingerprint

The photoacidity strength of the *pyranine–water–acetate* system is intermediate between that of pyranine in pure water and of pyranine–acetate in direct contact. In the first case, the sequential activation of low frequencies vibrational modes is necessary to the ESPT event, inducing a structural optimization between the proton donor–acceptor pair.^36^ More specifically, the photoactivated rings wagging and breathing modes provide the relative orientation of the proton donor–acceptor pair. These vibrational dynamics have been experimentally observed by FSRS experiments,^30,32^ and were recently found and analyzed by our computational approach.^36,53^ On the other hand, the direct ESPT from pyranine to acetate proceeds barrierless,^87^ suggesting that the low charge transfer degree of the pyranine after the excitation coupled to the deprotonated base in close proximity of the HPTS acid group, are the driving forces of the ESPT event. The presence of the water, connecting the pyranine and acetate as in the *pyranine–water–acetate* triad, leads to a barrier along the ESPT energy profile.^87^ This suggests that the vibrational activity detected in the first picosecond following the electronic excitation is responsible for the structural optimization required for the ESPT reaction to take place. Here, the connection between the excited state ESPT event and the underlying vibrational dynamics is established through the time-resolved vibrational analysis described in detail in section 2.2. The analysis allows one to extract generalized vibrational modes from molecular dynamics trajectories, even at low frequency (<600 cm^–1^) . Here, we focused on the key vibrational modes that in the time window of 1 ps seem to be important in promoting the reaction.

**Figure 4:**
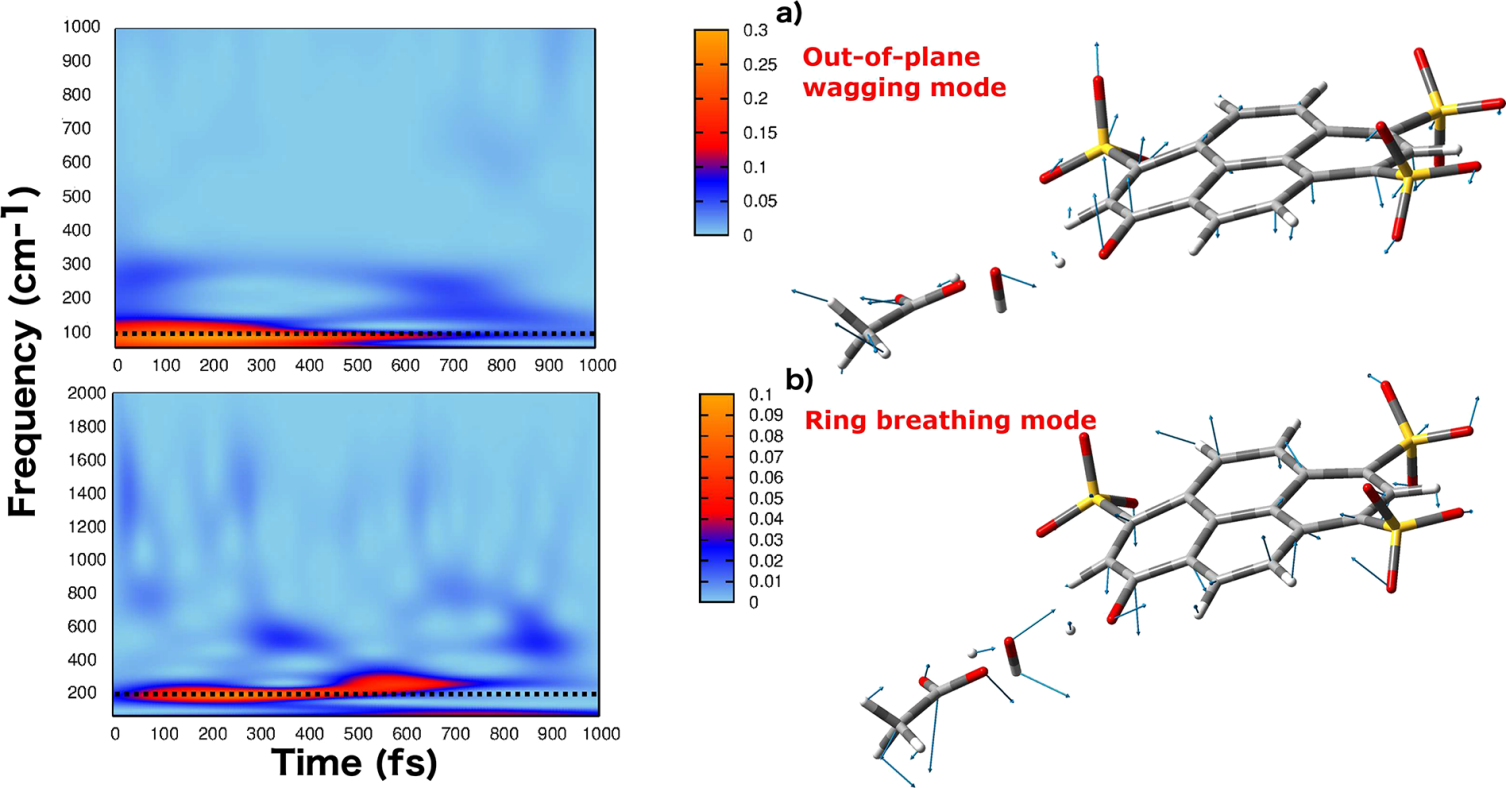
*Q* modes (right panels) and corresponding 2D wavelet power spectra (left panels). The color scale states for the intensity in arbitrary units. (a) Ring out-of-plane mode; the AIMD frequency is 110 cm^–1^, with an experimental value of 106 cm^–1^.^30^ (b) Ring breathing mode, with an AIMD frequency of 198 cm^–1^, with an experimental value of 195 cm^–1^.

In particular, we considered two vibrational low frequencies skeleton modes: the four-ring out-of-plane wagging and the collective breathing. From analysis of AIMD trajectories (see eqs 1 and 2), these modes are localized at 110 and 198 cm^–1^, respectively. The corresponding normal modes computed on the ground and excited state minima are reported in Figures S6 and S7. The 2D wavelet maps are given in Figure 4.

**Figure 5:**
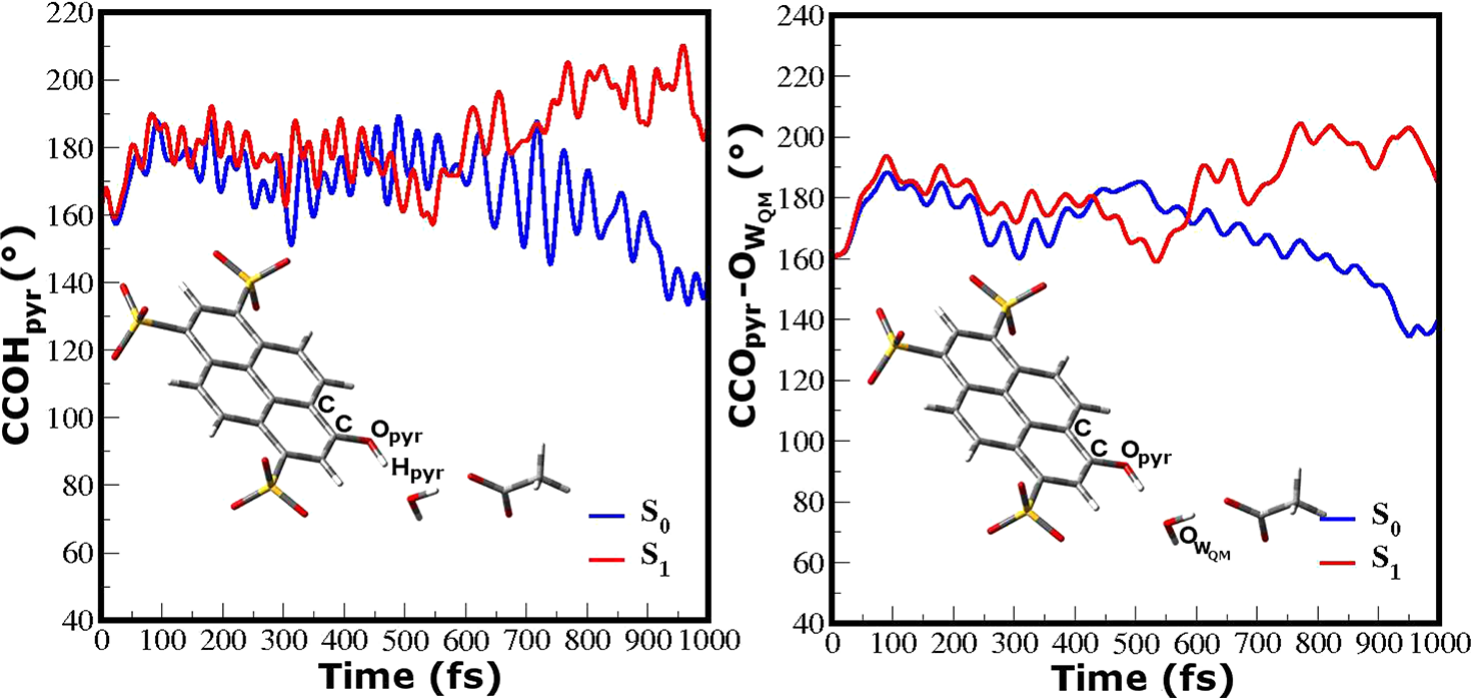
Comparison between the ground (blue lines) and excited (red lines) state behavior of the CCOH_*pyr*_ (left panel) and CCO_*pyr*_*–* (right panel) dihedral angles. The average values on the ground state are 167° and 168° for CCOH_*pyr*_ and CCO_*pyr*_*–* respectively, while on the excited state they oscillate around planar values (182° and 183°, respectively).

The out-of-plane wagging and the breathing are collective modes involving the whole nuclear skeleton. In both cases, a nice agreement with the experimental frequency^30^ is found (experimental frequencies of 106 and 195 cm^–1^ for wagging and breathing, respectively). The wagging mode is a ring out-of-plane movement. The 2D wavelet map of the wagging mode clearly shows the frequency at 110 cm^–1^ and the decay before 1 ps. The out-of-plane motion, mainly localized on the CCOH phenolic moiety, modulates the relative orientation of the pyranine phenolic group and the proton accepting water molecule. As evidence of the structural rearrangement of the pyranine in the excited state, we compare the time evolution of the CCOH_*pyr*_ and COO_*pyr*_*–* dihedral angles in the ground and excited states (Figure 5). These dihedral angles define the orientation and the planarity of the heavy atoms involved in the ESPT reaction. We can observe that their oscillations evolve around the planarity upon excitation (182° and 183° for CCOH_*pyr*_ and CCO_*pyr*_*–* respectively) with respect to the ground state (167° and 168° for CCOH_*pyr*_ and CCO_*pyr*_*–* respectively).

The breathing mode is, instead, an in-plane collective motion having a strong O_*pyr*_– intermolecular stretching component. The breathing motion corresponds to the stretching of the whole molecule and the approaching of the heavy atoms O_*pyr*_ and . The corresponding 2D wavelet map, in Figure 4 shows the contribution at 200 cm^–1^ associated with the breathing mode. This vibration appears immediately after the excitation and decays at about 700 fs, in nice agreement with the experimental time decay of 680 fs. The wagging and breathing modes were already identified for photoexcited pyranine in pure water, and it was found that within the time window of 1 ps they were essential to optimize the structural arrangement of pyranine–water before the ESPT event.^36^ Our findings suggest that this peculiar vibrational dynamics is characteristic of the photoexcited HPTS chromophore, regardless of the presence of the base in solution.

## 4 Conclusions

In this work we studied the photoinduced dynamics of the ESPT process involving the pyranine photoacid and acetate linked by one bridge water molecule. We used ab initio molecular dynamics in combination with an hybrid explicit/implicit model of solvation. Our findings suggest that the choice of the QM/MM partition dramatically affects the reactivity on the excited state. A clear correlation between the size of the QM/MM partition and the ESPT kinetic has been indeed found. Describing the solvation shells in proximity to the active site an MM point charge model is insufficient due to its inability to correctly polarize the reactive core and stabilize the reaction products and transition state. The ESPT reaction takes place only when the refined hydrogen bond network, in which the *pyranine–water–acetate* cluster is embedded, is taken into account at the fully QM level. The vibrational modes playing a key role in optimizing the structural arrangement of the chromophore and the proton acceptor have been also identified. The composition of the two main low frequency vibrational modes suggest that they are involved in the modulation of the ESPT reaction coordinates. In particular, the ring breathing (198 cm^–1^) supports the rearrangement of the intermolecular distances, while the out-of-plane wagging (110 cm^–1^) ruling the CCOH_*pyr*_ and CCO_*pyr*_*–* dihedral angles ensures the planarity of the proton donor–acceptor couple. The photoexcited pyranine in pure water solution shows the same vibrational fingerprint, as highlighted by experimental and theoretical investigations.^33,36^ While the acetate in solution speeds up the ESPT, the pyranine undergoes a structural rearrangement in order to optimize its interactions with the proton acceptor in a preparatory stage for the reaction. Although these low frequency vibrational modes are characteristic of the photoexcited pyranine, their activation and effects are common among the ESPT reactions, as observed for instance in the green fluorescent protein.^51,88^ The computational approach here adopted, which combines the extraction of normal-like modes from ab initio molecular dynamics simulations with a time-resolved vibrational analysis through the wavelet transform, confirms it to be a robust and general tool to unveil photoinduced relaxation and reactivity. The photoinduced nuclear dynamics of the photoinduced acid–base reactions are therefore described in terms of vibrational modes. This provides a direct comparison with spectroscopic experimental data and creates a direct link between the proton transfer reactivity and the underlying vibrational dynamics.

## Supporting Information Available

Harmonic frequencies computed on the ground and excited state minima, comparison of the ground and excited state dynamics, and frontier molecular orbitals associated with the bright electronic transition (PDF)
