# Supplementary material for: Water-Mediated Excited State Proton Transfer of Pyranine–Acetate in Aqueous Solution: Vibrational Fingerprints from Ab Initio Molecular Dynamics
Source: J Phys Chem A. 2021 Apr 26;125(17):3569–78. doi: 10.1021/acs.jpca.1c00692 (PMC8279639; doi:10.1021/acs.jpca.1c00692)
Supplement: Supplementary file 1 — jp1c00692_si_001.pdf [file jp1c00692_si_001.pdf]

Supporting information for: Water Mediated Excited State Proton Transfer of Pyranine-Acetate in Aqueous Solution: Vibrational Fingerprints from Ab-Initio Molecular Dynamics

Maria Gabriella Chiariello,† Umberto Raucci,† Greta Donati,† and Nadia Rega*,†,‡

†*Dipartimento di Scienze Chimiche, Università di Napoli Federico II, Complesso Universitario di M.S.Angelo, via Cintia, I-80126 Napoli, Italy*

‡*CRIB Center for Advanced Biomaterials for Healthcare, Largo Barsanti e Matteucci, I-80125 Napoli, Italy*

E-mail: nadia.rega@unina.it

Table S1: Harmonic and AIMD frequencies in cm^{-1} and kinetics (fs) of the principal vibrational modes. Harmonic frequencies are computed in the S_0 and S_1 minimum energy structure computed at B3LYP/6-31g(d,p)/C-PCM level of theory.

	Exp	Decay	Harmonic S_0	Harmonic S_1	AIMD S_1
4-ring out-of-plane wagging	106	1000	121	120	110
Skeletal in plane ring breathing	195	680	217	217	198

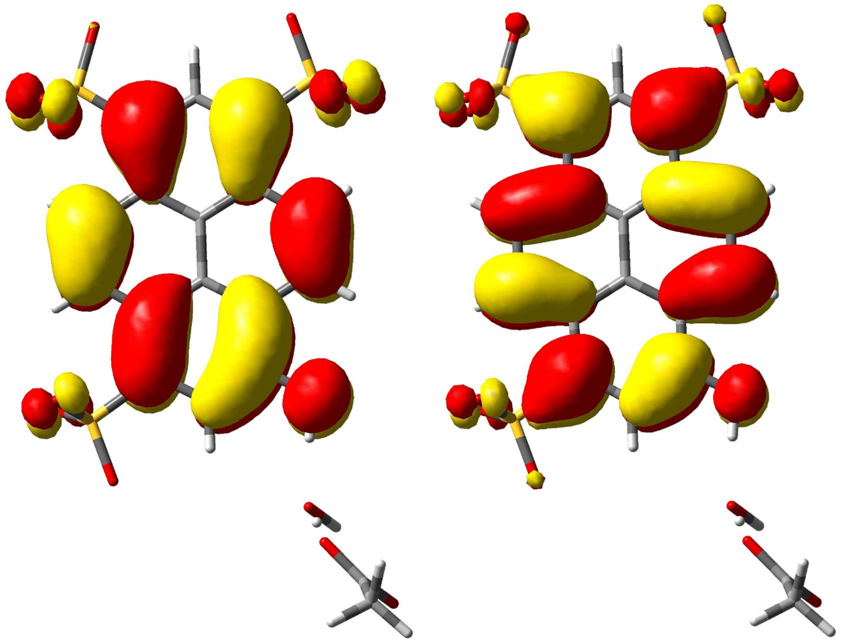

Figure S1: HOMO LUMO contours of HPTS chromophore computed at TD-DFT/B3LYP/6-31g(d,p)/CPCM level of theory on pyranine-water-acetate minimum energy structure. The vertical excitation is 398 nm.

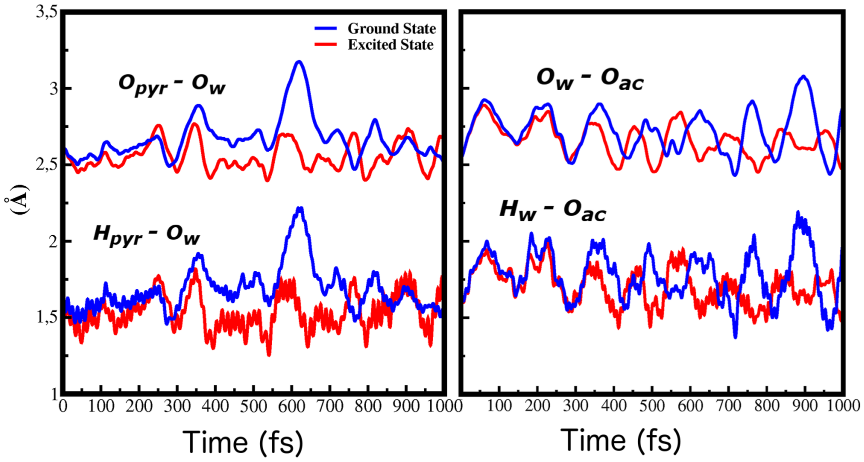

Figure S2: Comparison between the ground (blue lines) and excited (red lines) state behaviour of the OH_{pyr}, OH_w, H_{pyr}-O_w, H_w-O_{ac} parameters. The excited state trajectory has been collected using the QM/MM partition of the grond state.

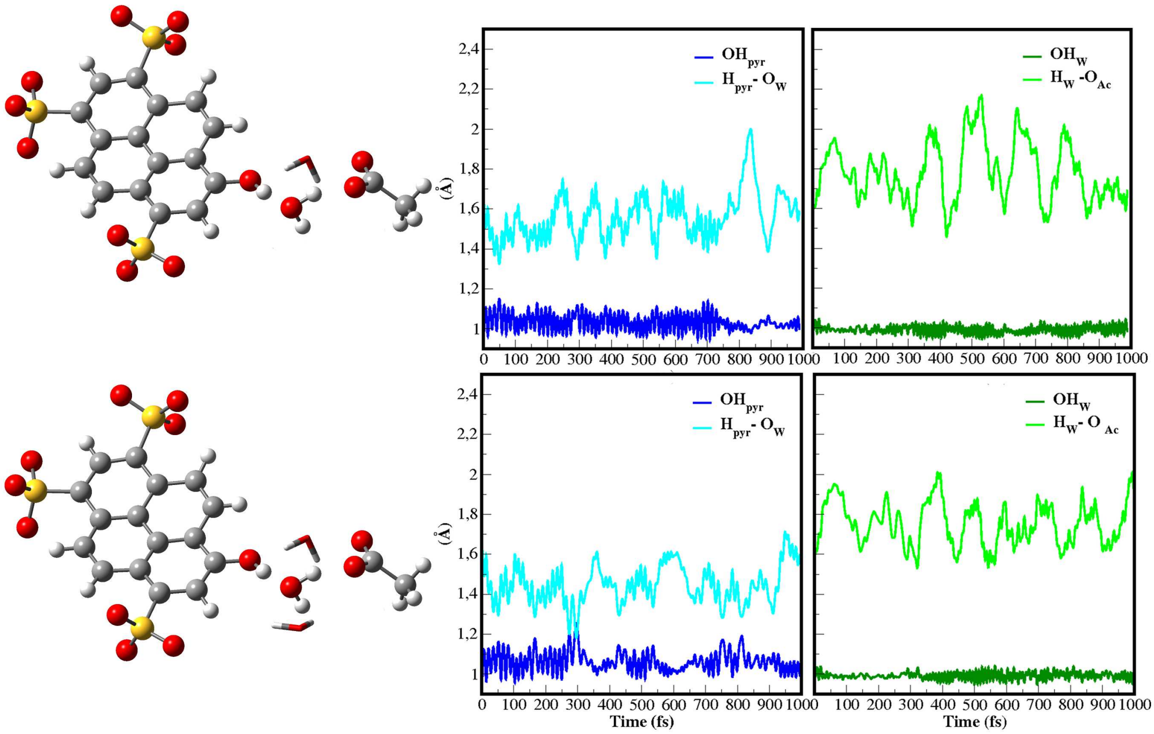

Figure S3: Excited state trajectories for two different QM/MM partitions. **Left panels:** One (up panel) and two (bottom panel) water molecules depicted in licorice representation are included in the QM region. **Right panels:** time evolution of the main structural parameters involved in the ESPT reaction OH_{pyr}, OH_w and H_{pyr}-O_w, H_w-O_{ac}.

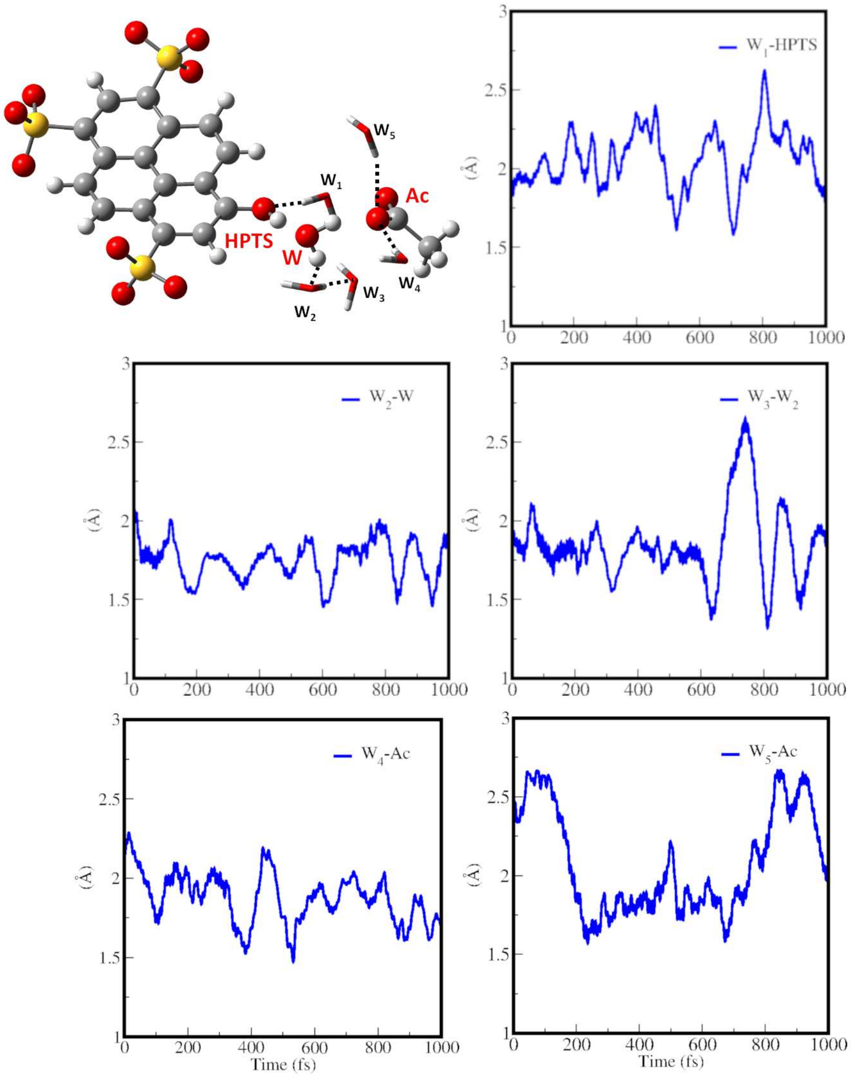

Figure S4: Time evolution (TRAJV) of the water molecules (W_1-W_5 depicted in the inset in the top left panel) belonging to the QM region. All the waters are H-bonded to the pyranine-water-acetate system in the 1 ps long excited state trajectory.

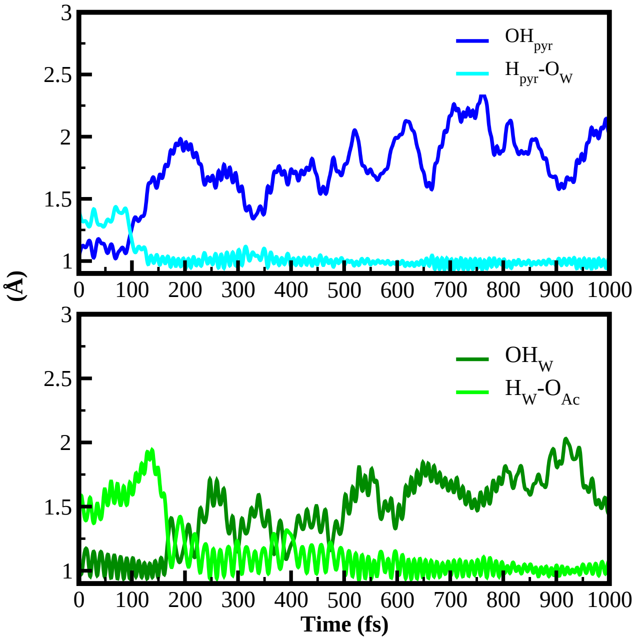

Figure S5: Excited state trajectory produced from a different starting configuration than TRAJI-V, but including five water molecules in QM region. The plot shows the time evolution of the main structural parameters involved in the ESPT reaction OH_{pyr}, OH_w and H_{pyr}-O_w, H_w-O_{ac}.

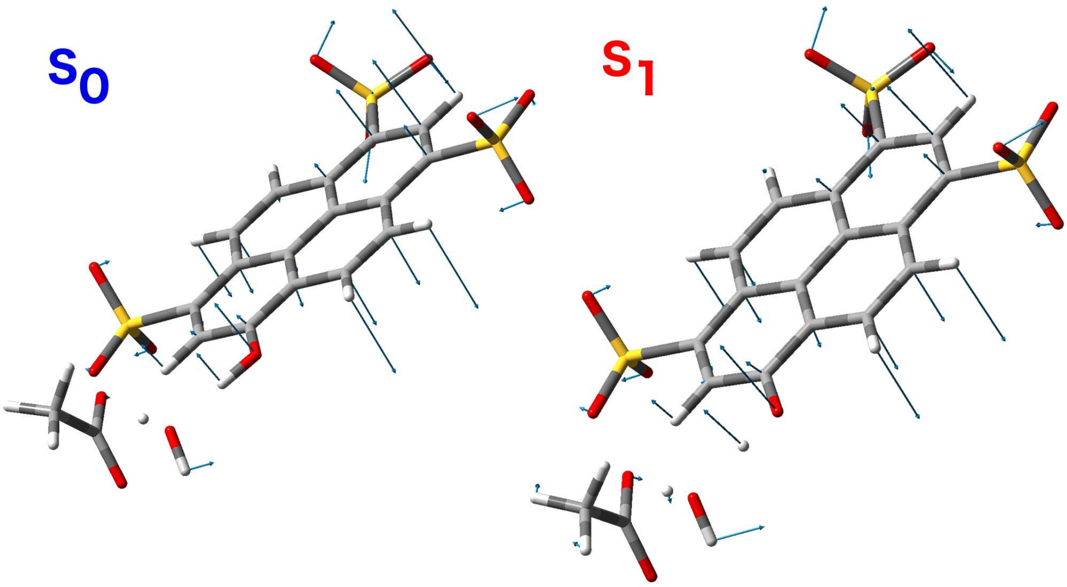

Figure S6: Composition of the ring out-of-plane wagging mode calculated in the a) S_0 (121 cm^{-1}) and b) S_1 (120 cm^{-1}) minimum.

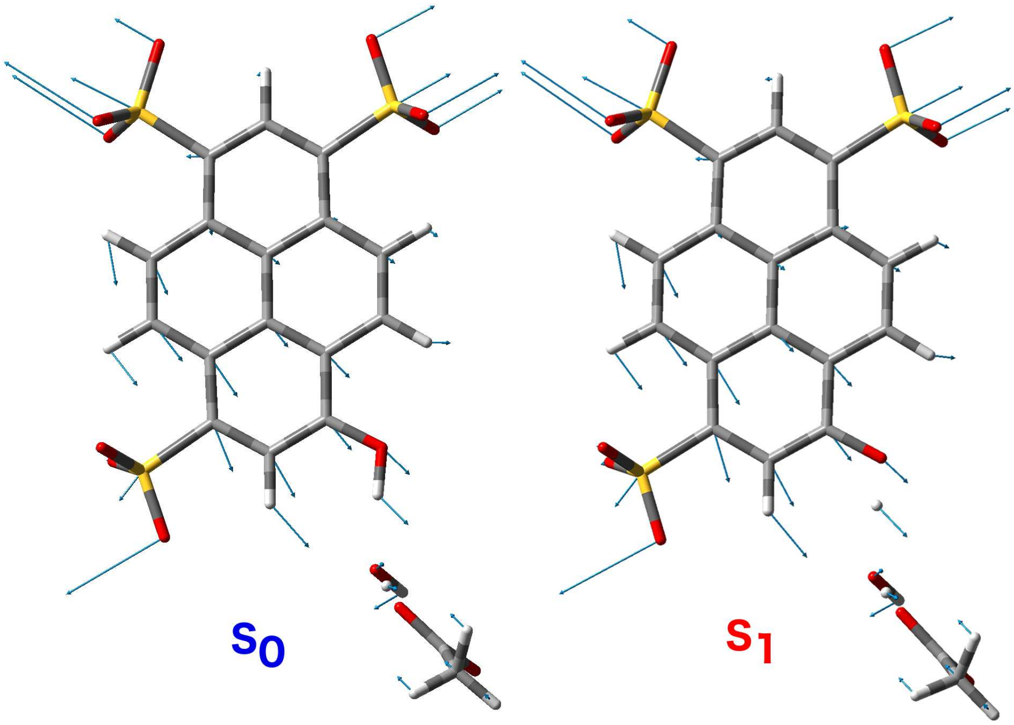

Figure S7: Composition of the skeletal ring breathing mode calculated in the a) S_0 (217 cm^{-1}) and b) S_1 (217 cm^{-1}) minimum.
